# Alignment-Free Design of Highly Discriminatory Diagnostic Primer Sets for *Escherichia coli* O104:H4 Outbreak Strains

**DOI:** 10.1371/journal.pone.0034498

**Published:** 2012-04-05

**Authors:** Leighton Pritchard, Nicola J. Holden, Martina Bielaszewska, Helge Karch, Ian K. Toth

**Affiliations:** 1 Information and Computational Sciences, James Hutton Institute, Dundee, Scotland, United Kingdom; 2 Cellular and Molecular Sciences, James Hutton Institute, Dundee, Scotland, United Kingdom; 3 Institute for Hygiene and the National Consulting Laboratory for Hæmolytic Uræmic Syndrome, University of Münster, Münster, Germany; University of California Riverside, United States of America

## Abstract

**Background:**

An *Escherichia coli* O104:H4 outbreak in Germany in summer 2011 caused 53 deaths, over 4000 individual infections across Europe, and considerable economic, social and political impact. This outbreak was the first in a position to exploit rapid, benchtop high-throughput sequencing (HTS) technologies and crowdsourced data analysis early in its investigation, establishing a new paradigm for rapid response to disease threats. We describe a novel strategy for design of diagnostic PCR primers that exploited this rapid draft bacterial genome sequencing to distinguish between *E. coli* O104:H4 outbreak isolates and other pathogenic *E. coli* isolates, including the historical hæmolytic uræmic syndrome (HUSEC) *E. coli* HUSEC041 O104:H4 strain, which possesses the same serotype as the outbreak isolates.

**Methodology/Principal Findings:**

Primers were designed using a novel alignment-free strategy against eleven draft whole genome assemblies of *E. coli* O104:H4 German outbreak isolates from the *E. coli* O104:H4 Genome Analysis Crowd-Sourcing Consortium website, and a negative sequence set containing 69 *E. coli* chromosome and plasmid sequences from public databases. Validation *in vitro* against 21 ‘positive’ *E. coli* O104:H4 outbreak and 32 ‘negative’ non-outbreak EHEC isolates indicated that individual primer sets exhibited 100% sensitivity for outbreak isolates, with false positive rates of between 9% and 22%. A minimal combination of two primers discriminated between outbreak and non-outbreak *E. coli* isolates with 100% sensitivity and 100% specificity.

**Conclusions/Significance:**

Draft genomes of isolates of disease outbreak bacteria enable high throughput primer design and enhanced diagnostic performance in comparison to traditional molecular assays. Future outbreak investigations will be able to harness HTS rapidly to generate draft genome sequences and diagnostic primer sets, greatly facilitating epidemiology and clinical diagnostics. We expect that high throughput primer design strategies will enable faster, more precise responses to future disease outbreaks of bacterial origin, and help to mitigate their societal impact.

## Introduction

The German O104:H4 outbreak of Summer 2011 was notable for several reasons. It represented in many ways a ‘worst-case’ scenario of an emerging pathogen with novel epidemiology and pathological characteristics, and is representative of the increasing burden of food-borne disease from contaminated fresh produce [1]. A number of outbreak isolates were characterised very soon after the outbreak by high-throughput sequencing technologies, and several draft genomes were placed in a public repository as part of a landmark open-source analysis [2]–[4]. The early availability of reference and isolate genome sequences greatly facilitated the identification of distinguishing sequence differences of outbreak isolates and elucidation of its evolutionary history [2], [5]. However, despite the intensive and timely application of this technology, and the contributions of many labs to this effort, practical difficulties in tracking the source of the outbreak resulted in significant social and political impact across several continents, highlighting a critical need for rapid generation of accurate diagnostics that can be used in the field, in public health outbreaks.

The ability of diagnostic techniques such as real-time quantitative PCR (qPCR) to deliver sensitive and quantifiable results is dependent on the availability of primer sets that distinguish a target organism or organisms from non-target organisms. Typically, design of discriminatory primer sets is rationally guided in the sense that primers are chosen to amplify genes or other defined sequences demonstrated to be common to target organisms but divergent, or absent, in non-target organisms [6], [7]. Frequently used sequences include intergenic transcribed spacer regions, ribosomal DNA, ‘housekeeping’ genes and virulence genes, e.g. Shiga-like toxins for *E. coli* [8]–[11]. Several high-throughput variants of this approach to diagnostic primer design have been described, and these may be characterised as the identification of potentially discriminatory sequences, followed by primer design against those sequences [12]–[17]. Here we demonstrate an alternative, alignment-free strategy for primer design that exploits incomplete and unordered draft genome sequences to identify candidate primer sets that discriminate between arbitrary subgroups of sequenced bacteria, without the need to pre-screen for discriminatory sequence prior to primer design. This approach can be characterised as the bulk design of primers to all input genome sequences, followed by classification of the discriminatory ability and specificity of those primers to predefined groups of input sequences. The outputs of the strategy are the sets of primers that are specific to each predefined group of sequences. The alignment-free strategy avoids limitations resulting from the need to identify a conserved signature of significant length and common to all targets, prior to primer design, and enables the simultaneous design of primers to discriminate between several (potentially overlapping) groupings of sequences.

The primers designed using this strategy are intended to be highly discriminatory, and to separate, where possible, closely related strains or isolates. Traditionally, methods used in a clinical diagnostic setting first identify bacteria to species level using microbiology techniques (typically selective agar and biochemical tests), followed by discrimination to the serotype level (e.g. by serotyping agglutination tests). For epidemiological analysis, isolates will typically be compared in more detail using techniques that exploit changes at the DNA level, most usually of ‘housekeeping’ genes. Such techniques would include multi-locus sequence typing (MLST) and multi-locus enzyme electrophoresis (MLEE) [18], [19] although, in general, these methods do not provide a sufficient level of discrimination to distinguish closely related isolates. Instead, techniques based on variable regions of DNA, such as pulsed field electrophoresis (PFGE), random amplified polymorphic DNA (RAPD) or multi-locus variable-number tandem-repeat (VNTR) analysis are typically used [20], [21]. Over and above these typing techniques, pathogenicity factors are also frequently included in the identification, e.g. the Shiga toxin type for VTEC [22].

We used our design strategy to generate several primer sets that discriminate *in silico* between outbreak isolates of *E. coli* O104:H4 and other *E. coli* isolates, including the historical and serotypically identical hæmolytic uræmic syndrome (HUSEC) *E. coli* HUSEC041 O104:H4 strain. We validated these potentially discriminatory primer sets against a bank of 21 ‘positive’ *E. coli* O104:H4 outbreak and 32 ‘negative’ non-outbreak EHEC isolates *in vitro*. Our results showed that individual primers exhibited 100% sensitivity and 82–94% specificity for outbreak isolates, with false positive rates of between 9% and 22% for non-outbreak isolates. However, a minimal combination of two primers was able to discriminate between outbreak and non-outbreak *E. coli* isolates with 100% sensitivity and 100% specificity.

## Materials and Methods

### Genome sequences

Eleven draft assemblies of nine *E. coli* isolates sampled from the 2011 German O104:H4 outbreak were downloaded from the *E. coli* O104:H4 Genome Analysis Crowdsourcing site (https://github.com/ehec-outbreak-crowdsourced/BGI-data-analysis/wiki; see Supplementary Methods S1), including two assemblies for each of the TY2482 and LB226692 isolates [2], [4]. A single pseudochromosome sequence was compiled where necessary for each draft sequence by concatenating contigs (in arbitrary ordering and orientation) using a spacer sequence [23]. These sequences constituted the ‘positive’ sequence set for primer set generation.

A set of 31 completely sequenced *E. coli* and *E. fergusonii* genomes were downloaded from GenBank (ftp://ftp.ncbi.nlm.nih.gov/genomes/Bacteria/) for use as the ‘reference’ or ‘negative’ sequence set during primer set generation. Accession numbers for all sequences are given in Supplementary Methods S1. Additionally, all refseq_genomic database sequences beneath the *Escherichia* taxonomic level (TaxID: 561) at the NCBI on 6/7/2011 were used as the online screening database.

### Primer prediction

**Figure 1:**
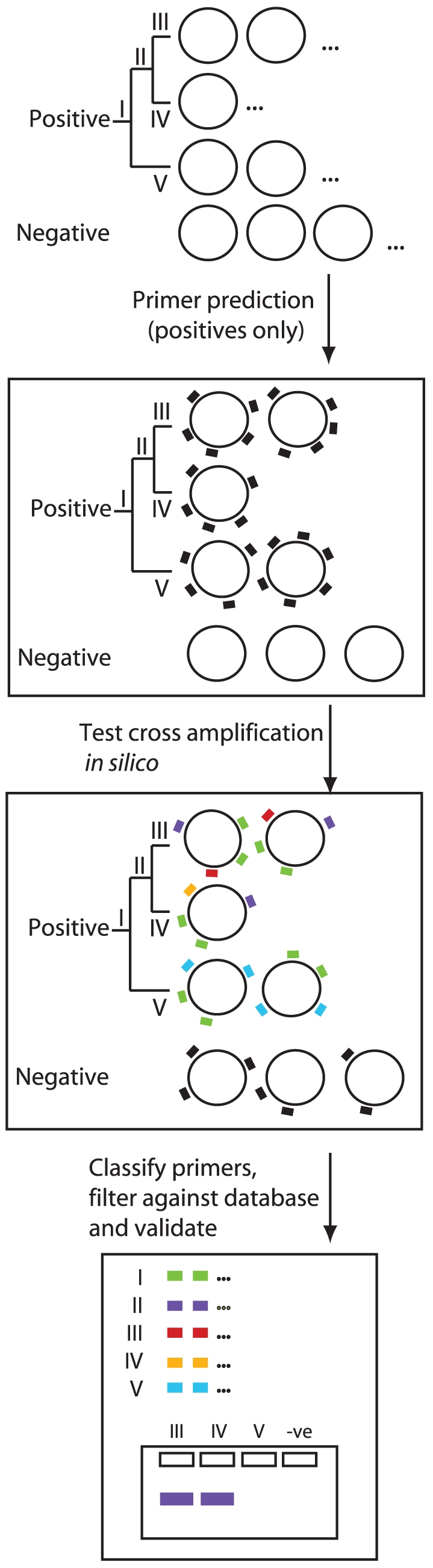
Schematic diagram of the primer design process. A training set of whole (complete or draft) genome sequences is divided into positive and negative sequence groups. Members of the positive sequence group are placed into classes as appropriate (here as classes I–V on the basis of a nested hierarchical relationship). Primer sets are designed to all positive sequences in bulk (>1000 primer sets, black markers), and tested for cross-hybridisation *in silico*. Primer sets that amplify only members of a prescribed class (indicated by coloured markers, one for each class; black markers indicate non-specific primers) but do not amplify negative examples are retained as being potentially diagnostic of that class. Predicted discriminatory primers are validated against bacterial isolates that were not part of the training set. An expected mock PCR result is indicated for primers specific to group II against individual samples belonging to classes II, IV and V. A detailed description of the method is given in Supplementary Methods.

**Figure 2:**
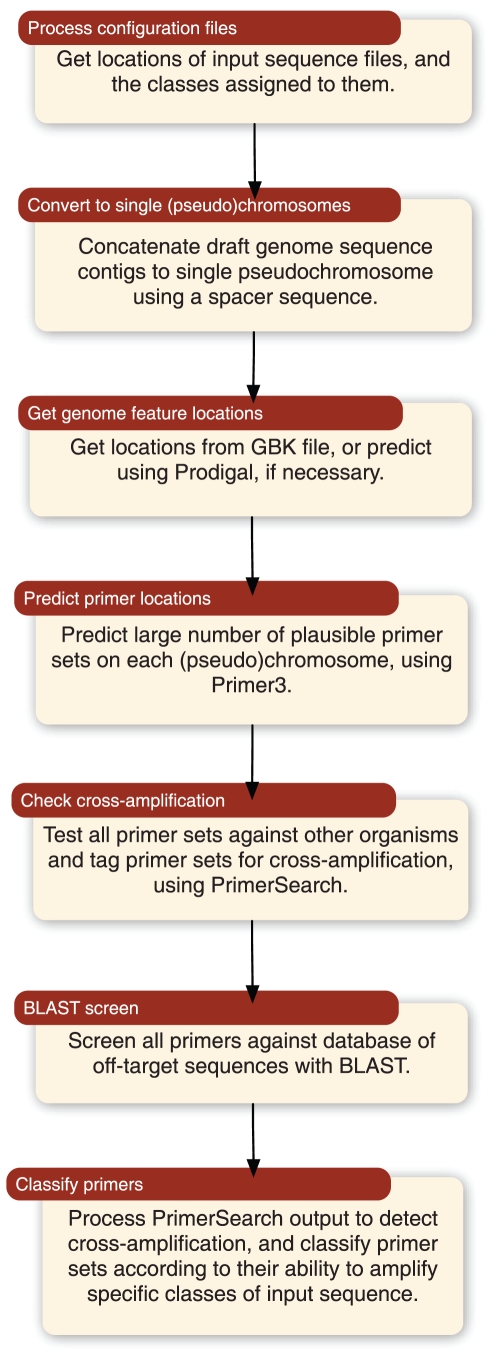
Flowchart of the primer design process. The locations of input training set sequence files, and their classifications, are read from a configuration file. Input sequences comprising several smaller sequences (e.g. contigs of a draft genome) are concatenated using a spacer sequence. Locations of coding sequences (CDS) are obtained from a GenBank file if available, or predicted using a genecaller. A large number (>1000) of primers is then designed to each input sequence. Primers that lie within CDS are tested *in silico* for their ability to cross-amplify other members of the training set, and compared against a larger set of off-target sequences to discard non-specific primers. The surviving primers are classified according to their ability to amplify specific classes of sequence from the training set. A more detailed flowchart of the pipeline is given in Supplementary Figure S1.

Diagnostic primers were designed to the genome sequences indicated above using a computational pipeline that implements the design strategy outlined schematically in Figures 1 and 2. This strategy can be implemented in several ways, using a choice of broadly equivalent software tools. Our specific implementation is described in detail in Supplementary Methods S1, and in Supplementary Figure S1. The Python scripts and configuration file used for this study are available at https://github.com/widdowquinn/find_differential_primers.

Our pipeline takes as input a set of whole or draft genome sequences. Each input sequence is assigned one or more labels. These labels (groups I-V in Figure 1) denote membership of a class, and may be based on any criterion or grouping for which one wishes to generate a diagnostic set of primers. An input genome sequence may be assigned multiple labels, indicating different groups to which one might wish to design diagnostic primers. This permits members of any class to be further subclassified, for example as a nested hierarchy to represent taxonomic assignments in which individual sequences may be labelled with both the name of the genus and the species (and pathovar, biovar or other subclass; Figure 1). This enables simultaneous design of primers that distinguish between multiple potentially overlapping groups of sequences, such as sets of primers that distinguish between bacterial genera, and (distinct) sets of primers that distinguish between species within a single genus.

A large number (typically 1000 or more, for a bacterial chromosome) of plausible primer sequence sets, compliant with thermodynamic or structural parameters specified when running the pipeline, such as melting temperature (T_m_) or 3′ GC content, is designed to each input genome sequence. These primer sets are expected, by definition, to amplify a region of the genome to which they were designed. The predicted primer sets may then be filtered to exclude those that lie wholly or partially outwith gene coding regions, so to focus on genomic regions that are more likely to be evolutionarily stable. The surviving primer sets are tested *in silico* for their ability to amplify each of the other input genome sequences. Primer sets are retained as being potentially diagnostic of a class of sequences only if they are predicted *in silico* to amplify a similarly-sized amplicon product from all genomes from the input set which share that label, but not from those genomes that do not possess the label. These primers may then be screened against a larger set of sequences that do not belong to the predicted class, and discarded if they exhibit significant sequence similarity. The primer sets that survive this screen are the output of the pipeline, and are considered to be potentially diagnostic of members of the class, pending experimental validation.

For this study, our ‘positive’ sequence set contained eleven draft assemblies of nine *E. coli* O104:H4 German outbreak isolates downloaded from the *E. coli* O104:H4 Genome Analysis Crowd-Sourcing Consortium website [2], [4] labelled as ‘outbreak O104:H4’, and the ‘negative’ genome set comprised 69 *E. coli* chromosome and plasmid sequences downloaded from the public databases at NCBI [24],[25], labelled as ‘not outbreak O104:H4’. Contigs from the draft bacterial genomes assembled by HTS were combined in arbitrary order into a single pseudochromosome sequence using a standard spacer sequence [23].

One thousand thermodynamically plausible primers were predicted across the whole genome or pseudochromosome for all members of the positive ‘outbreak O104:H4’ sequence set using ePrimer3 [26], [27]. To save time, primers were not designed to the ‘negative’ genome set. To enhance the expected evolutionary stability of primer sets, predicted primers that did not amplify within predicted coding sequences (CDS) on a chromosome were discarded. CDS prediction was carried out using the Prodigal [28] genecaller where required, otherwise existing CDS annotations from public annotations were used. The primer sets located within predicted CDS were tested *in silico* for their ability to produce amplicons of a desired size range from all members of the positive and negative sequence sets using PrimerSearch, to identify potential cross-amplification [27]. We retained only primer sets that amplified sequences of approximately 100 bp length from all members of the ‘outbreak O104:H4’ set, but did not amplify members of the ‘negative’ sequence set. The surviving discriminatory primer pairs and their amplified sequences were filtered again using BLAST [29] to exclude those primers that may not be specific to the targets on the basis of sequence similarity to the set of *E. coli* sequences contained in the NCBI RefSeq database [25]. On a desktop machine (8-core Mac Pro, 32GB RAM, OSX 10.6) the entire pipeline took approximately 90 hours, of which all but two hours were spent on the PrimerSearch step for detection of cross-amplification.

### Primer evaluation

The optimum annealing temperature for the complete primer set was first determined to be 58°C, using the sequenced E. coli isolate LB226692 as a positive control between 54°C and 58°C. Group1 (outbreak) and group 2 (non-outbreak) bacterial isolates were screened with each primer set under the determined optimum conditions. PCR reactions of 25 ml contained 500 nM of each primer pair, 800 mM dNTPs, 1× buffer solution, 1× enhancer solution, 2 mM MgCl2, 0.5 units/µL peqGOLD Taq polymerase (peqlab Gold PCR kit, peqlab ltd., Germany) and 1 ml DNA (∼100 pg). Standard cycling conditions were used: one cycle of 94°C for 5 minutes; 30 cycles of 94°C for 30 seconds, 58°C for 1 minute, 72°C for 1 minute; one cycle of 72°C for five minutes. Products were resolved on a 1% agarose gel in tris-acetate-EDTA (TAE) buffer at 1× concentration, alongside a 100 bp ladder. The images were captured under standard conditions in a gel documentation system (AlphaImager, Labtech) at 300 dpi and saved in TIFF format. Image manipulations (combination of multiple gel results into a single figure) were carried out using Adobe Illustrator.

## Results

### Primer design

Seven non-degenerate primer sets were predicted using the strategy outlined in Figure 1 to be capable of discriminating between the positive outbreak and negative non-outbreak *E. coli* sequence sets *in silico* (Supplementary Table S1). On filtering these primers against the RefSeq database [30], three primer sets (0237, 0396 and 0781) were found to have sequence similarity either to the *impB* gene on the pEC_Bactec β-lactamase plasmid or a hypothetical protein in at least one other recently sequenced non-outbreak (O127:H6) *E. coli* isolate. The remaining four primer sets (three overlapping: 0220, 0376, and 0393; and 0901) amplify two regions within a putative prophage gp20 transfer protein. The predicted amplicon for the 0393 primer set encompassed only sequence that would have been amplified from a combination of 0220 and 0376 pairs. Hence, of these sets, only 0393 was carried forward for validation.

Five primer sets (0237, 0393, 0396, 0781, 0901) were taken forward for experimental validation of their discriminatory ability under standard PCR conditions. Validation took place against two groups of pathogenic *E. coli*: group 1 was a ‘positive’ set comprising 21 clinical O104:H4 isolates from the recent German outbreak, collected from Universitätsklinikum Münster [31]; group 2 was a ‘negative’ set and comprised 28 HUSEC isolates from the Universitätsklinikum Münster collection [32], and four additional EPEC and/or HUSEC isolates (Supplementary Table S2). The sequenced outbreak isolate LB226692 [3] served as a positive control.

**Figure 3:**
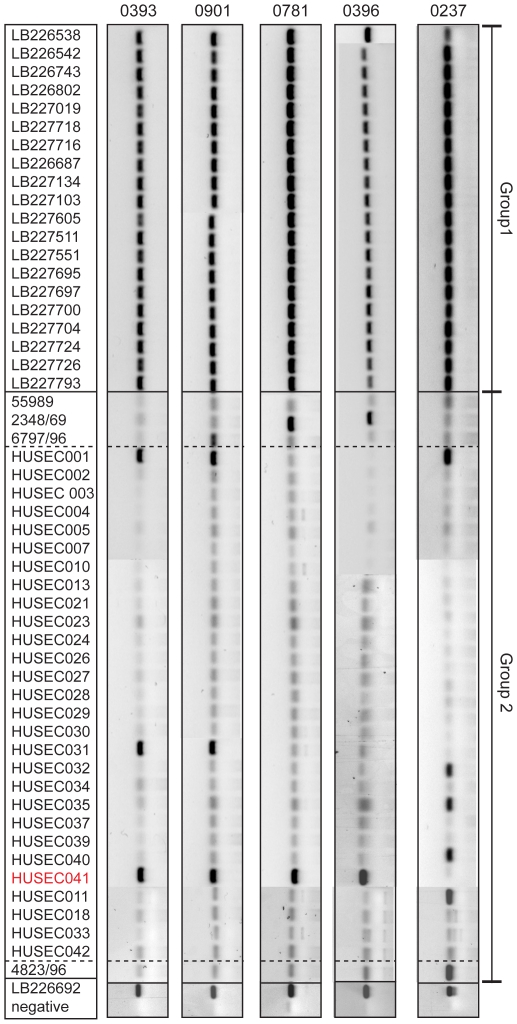
PCR amplicons from the *E. coli* clinical isolates, grouped by primer set. Composite (negative) images from multiple agarose gels have been aligned against the matching isolate designation (left hand column) and below the primer names (top row). The image has been split so that Group 1 isolates are located in the top section, Group 2 isolates in the second and the positive and negative controls at the bottom. The HUSEC isolates within Group 2 are bounded by dashed lines and *E. coli* HUSEC041 indicated in red font.

Each of the primer sets exhibited 100% sensitivity for the 21 ‘positive’ outbreak isolates in group 1, with 82–94% specificity, and false discovery rates between 9% and 22% (Figure 3; Table 1; Supplementary Table S3). It was possible, using a minimal combination of two primer sets (e.g. 0393 and 0237) to discriminate between the 21 outbreak O104:H4 isolates and all 32 non-outbreak *E. coli* isolates in our validation dataset with absolute sensitivity and specificity. Thus, on the basis of draft bacterial genome sequences, our strategy rapidly generated a bank of PCR primers that were able to discriminate absolutely between the recent German outbreak *E. coli* O104:H4 isolates and other *E. coli*, including a historical isolate with the same serotype.

**Table 1:** Statistical performance metrics for diagnostic primer sets.

Primer set:	393	901	781	396	237
TP:	21	21	21	21	21
FP:	3	4	2	2	6
TN:	30	29	31	31	27
FN:	0	0	0	0	0
Sensitivity (recall):	1.00	1.00	1.00	1.00	1.00
Specificity:	0.91	0.88	0.94	0.94	0.82
PPV (precision):	0.88	0.84	0.91	0.91	0.78
FPR:	0.09	0.12	0.06	0.06	0.18
FDR:	0.13	0.16	0.09	0.09	0.22
F-measure:	0.93	0.91	0.95	0.95	0.88

The confusion matrix counts (TP: true positive; FP: false positive; TN: true negative; FN: false negative) derived from the experimental validation tests against unseen isolates of *E. coli* are presented. No false negatives were identified by any individual primer set. Derived performance measures are also indicated (PPV: positive predictive value; FPR: false positive rate; FDR: false discovery rate; F-measure: 2×recall×precision/(recall+precision)). All primer sets amplify all positive examples, and have specificity between 82–94%, with 9–22% false discovery rate.

## Discussion

The *E. coli* O104:H4 outbreak in the summer of 2011 caused significant loss of life and incurred financial and political consequences affecting countries on more than one continent. At that time, the discriminatory primer set for *E. coli* O104:H4 comprised a bank of four primers, targeted to specific functional sequences: the O-antigen (*rfbO104*), the flagellar locus (*fliC H4*), the tellurite resistance gene (*terD*), and the toxin gene *stx2*, which was unable to discriminate the historical HUSEC041 isolate from the outbreak strain [3], [22], [33]. The primers designed in this study improve on this, as fewer primers are required to distinguish with greater specificity between outbreak and non-outbreak strains, including the historical HUSEC041 O104:H4 strain.

At the time of writing, the *E. coli* O104:H4 outbreak isolate reservoir remains to be determined, but our primer sets support recent conclusions that the sequenced outbreak *E. coli* LB226692 isolate belongs to the HUSEC complex [2], [3], [5], [33]. HUSEC041 was unique amongst our negative set of validation bacteria in that a positive result was seen with all primer sets other than 0237. This result is indicative of differences in the TEM-1 plasmid harbouring CTX-M-15 on the Tn3 transposon. It is notable that primer set 0237 also amplifies four HUSEC isolates and two additional *E. coli* O104 isolates (Figure 3; Table 1).

Rapid generation of draft genome sequences for bacterial pathogens involved in disease outbreaks presents an opportunity for the generation of powerful discriminatory diagnostics that has not previously been available. There have been several proposals for strategies to exploit such data for diagnostic primer design, each of which involves the pre-identification of sequence regions common to a set of target sequences, and the design of primers that amplify those regions [12]–[17]. A recent such study has used draft genome sequences to design discriminatory primers to outbreak O104:H4 isolates [17]. Our strategy avoids limitations of these methods in the following key ways.

Methods such as KPATH [16] that depend on the construction of consensus regions from whole genome multiple alignments are particularly computationally intensive due to the whole genome alignment step. This has a heavy scaling penalty when more than a small number of genomes are aligned that is avoided by methods such as TOFI, Insignia, and ssGeneFinder, by the use of pairwise genome alignment or progressive sequence subtraction, which is computationally more efficient [12], [13], [15], [17]. However, as has been noted elsewhere [12], TOFI and Insignia are limited in their application, by design. Insignia restricts the design of primers to sequences that are already present in the server's database, and TOFI is only capable of designing features for microarray-based diagnostic assays [13], [15].

The ssGeneFinder package uses progressive alignment and subtraction of regions of similarity to off-target sequence in order to restrict primer design to potentially discriminatory regions of the target sequences [17]. Similarly, TOPSI identifies common regions of target genomes that are not shared with off-target genomes to design primer sequences that are predicted to amplify only target sequences [12]. By contrast, our strategy is alignment-free in that no genome alignment takes place prior to primer design. This has two advantages over pairwise alignment. It eliminates a computationally intensive step from the pipeline, and it enables the simultaneous design of primers that discriminate between several subclasses of input sequence.

For example, consider a set of input sequences that comprises several isolates of two species within the same genus (‘*Bacterium alpha*’ and ‘*Bacterium beta’*), and a set of related isolates that are not from that genus (‘*Anabacterium* spp.’). In order to design primer sets that discriminate at the genus level and positively identify each species, a method that relies on genome alignment to generate a consensus sequence specific to each target group would need to be applied three times: once to generate primers specific to the genus, and twice more to generate primers that target each species. By avoiding pairwise genome alignment and generating a single large set of thermodynamically plausible and potentially discriminatory primers (a relatively cheap process, computationally) we are able to test each primer for the ability to discriminate between arbitrary groupings of the input sequences on a single application of the method. We have applied our strategy successfully to the draft genomes of 25 bacterial isolates for simultaneous design of primers that discriminate between six species within a single bacterial genus, and that distinguish members of the genus from non-members of the genus, in a single pass (LP, unpublished data).

Alignment-based methods typically require substantial similarity between target sequences in order to raise potentially discriminatory primer predictions. This is not the case for our alignment-free method, which may help eliminate another limitation of those methods: the difficulty of designing signatures for viral genomes. These are small and highly variable, posing a particular problem for whole genome alignment [12], [14]. We have as yet no data for the design of primers to viral sequences using our approach, but a comparison with alignment-based methods would potentially be informative, as for short sequences our strategy encompasses the possibility of exhaustive design and explicit *in silico* cross-amplification testing of thermodynamically plausible primers. This may reveal diagnostic primer sets, or panels, that would not be found when focusing on aligned regions of high sequence similarity.

The central limitation of alignment-based primer design methods is their reliance on the presence of a conserved signature of sufficient length to which primers may be designed. If such a region is shared by some, but not all, sequences in the target set, that region is excluded from consideration for primer design. This is a particular issue for the design of primer sets to draft or incomplete bacterial genome sequences, as regions of potentially high discriminatory capacity may be eliminated because of a single low-quality or incomplete sequence [12]. Our alignment-free approach does not exclude these regions so that, where it arises that one or more incomplete or low-quality sequences would prevent alignment methods from identifying a consensus region for primer design, it would still be possible to identify primers, or sets of primers, that amplify only members of the target sequence set.

Our implementation of the primer design strategy for this study suffered from known scaling issues associated with the PrimerSearch software chosen for the *in silico* cross-validation prediction step. Of the total 90 hour running time, 88 hours was spent on this calculation (see Supplementary Figure S2). Our primer design strategy is modular, and it would be possible to exchange the PrimerSearch package for any equivalent, faster approach that identified potential cross-amplification. We identified no direct replacement for this package during the study, but alternative algorithms based on established approaches to short read mapping (e.g. [34]–[36]), or that exclude candidate primer sets from consideration at the first indication that they amplify an off-target sequence, or fail to identify any possible target class, may result in a shorter run time at this step.

Primer validation *in vitro* against isolates that were not used in the primer design process is an essential step to establish the efficacy of the designs, regardless of the primer design strategy. Ho *et al.*, who also designed discriminatory primers to *E. coli* O104:H4 using ssGeneFinder, described three primer pairs that were validated *in vitro* on a collection of 65 E. coli isolates, 25 culture-negative stool samples, and 7 environmental soil samples, but the nature and pathogenic or clinical relevance of those isolates is otherwise unclear [17]. We applied a more comprehensive and rigorous validation test to our primer sets, using a robust bank of serotyped outbreak and non-outbreak clinical *E. coli* isolates, and were able to establish that our predicted primers could be used to discriminate between isolates from different outbreaks, but with the same serotype. Some differences in the intensity of the PCR bands were evident, although the vast majority were strongly positive, indicative of a high concentration of amplified product. There was evidence of low specificity binding, in particular with primer set 0781. Although it is not always possible to circumvent such issues with PCR, and quantitative (q)PCR could be used to define the response more precisely, our use of conventional PCR showed clear differences in the ‘positive’ and non-specific signals that were deemed ‘negative’ (Fig. 2).

PCR-based methods for diagnostic identification of bacterial isolates on a large scale are likely to remain a robustly useful technology, even in a potential future of very cheap bacterial genome sequencing. Arguably, high-throughput sequencing is already the most economic approach to typing a bacterium and, given current trends of increasing pervasiveness and falling cost of the technology, this is unlikely to change. However, high-throughput sequencing technology is not yet sufficiently widespread or cheap to enable rapid sequencing of each bacterial isolate, every suspected case of infection, or all samples collected from every potential source of infection. In situations such as the tracking of a disease outbreak, or prophylactic screening, there will remain a place for molecular diagnostic tests on economic grounds alone, as costs (and downstream analysis effort) for diagnostic PCR test of a bacterial sample are likely to remain favourably comparable that of sequencing for some time to come.

The increasing global footprint and reducing cost of high-throughput sequencing is, however, an extremely important component of the potential future applicability of our strategy for primer design, which rests on the availability of a sufficient number of well-chosen example genome sequences. The *E. coli* O104:H7 outbreak was highly unusual in this regard, as there already existed a large number of publicly available reference genome sequences, and high-throughput sequencing was rapidly applied to generate a reasonable number of genomes for outbreak isolates. This fortunate situation might not have occurred in 2011 for an outbreak of any other pathogenic bacterium that had not been the focus of a great deal of historical sequencing effort. We expect that future outbreaks of bacterial contamination and infection will be in a better position to exploit modern sequencing technologies, both for outbreak strains and for reference strains held in bacterial collections. A future $10–$50 bacterial genome sequence may in this way underpin the design of extremely inexpensive and rapid, but robust, PCR-based diagnostic tests for application ‘in the field’.

By our method, the predicted specificity of diagnostic PCR primers is determined by the size, coverage and composition of the training and screening sequence sets used to generate them, and by the nature of the differences between the classes one aims to distinguish. The purpose of diagnostic primer sets such as those designed in this study is to classify previously unseen samples, ideally with a quantifiable degree of confidence, but classifications can be drawn along arbitrary lines that may or may not coincide with taxonomic class or biochemical capability. Where the differences between classes are such that they cannot be detected using PCR primers, such as for genomes with extremely low sequence diversity, the approach described here is inappropriate. Previous applications of our primer design strategy have, unsurprisingly, failed when attempting to discriminate between nearly clonal isolates of the same bacterial species (LP, unpublished data).

Horizontal gene transfer is a key contributor to the evolutionary dynamics of pathogenic bacteria [1], [37], [38], and has particular implications for the ability to generate diagnostic primers specific to a novel outbreak. Where an emergent pathogen has obtained virulence factors in this manner, it may be distinguished from its close phylogenetic relatives by primers that amplify the laterally acquired region. However, such primers will likely produce false positives against the bacteria from which the region was acquired, if applied in isolation. Careful choice of training classes, screening database, validation strains, and the use of panels of several diagnostic primers (perhaps specific to different classes) can help overcome this issue. On a practical level, although all primer sets designed in this study were predicted to be specific to outbreak O104:H4 isolates, we found in experimental validation that a combination of at least two primer sets was required for absolute specificity. (Cross)-validation is a key step in the production of any classifier, and is especially important here, where the background variation is likely to be great in comparison with the sequences chosen for the training and primer discovery step.

Training and screening sequence datasets are necessarily incomplete and biased, as they cannot reasonably contain sequences for every isolate that is known or may be encountered in the field, and nor can they encompass the full range of variation across bacteria. The rate of addition of complete and draft sequences to the public databases is however very rapid, and offers an opportunity for continued monitoring of primer specificity in silico as new sequences become publicly available. Identification of potential off-target amplification against newly published sequences *in silico* may be automated to flag a need for revision of primer specificity, and possible primer redesign. Subsequent to the experimental validation of the predicted primers in this study, the complete sequence of a novel *E. coli* isolate (UMNF18; GenBank CP002890.1) that was unavailable during our training and validation process was entered into public repositories. This genome exhibits *in silico* potential off target amplification by the four primers that target the gp20 transfer sequence (0220, 0376, 0393, and 0901), highlighting the necessity for continued monitoring of primer specificity as new sequences become available.

Our results demonstrate that timely sequencing of representative isolates of disease outbreak bacteria enables high throughput diagnostic primer design that exceeds the discriminatory capabilities achieved using restricted sets of known ‘housekeeping’ genes, or contributors to virulence, that typically characterise molecular assays. In combination with the genome-wide alignment-free diagnostic primer design strategy described here, the rapid sequencing of representative O104:H4 outbreak isolates enabled precise molecular diagnostics to be designed that were targeted directly to the outbreak isolates.

## Supporting Information

Figure S1 Flowchart of the primer design process. Flowchart indicating steps implemented in the find_differential_primers.py Python primer prediction script, and the effects of major command-line options.
(EPS)

Figure S2 Flowchart of the primer design process for O104:H4 specific primers, indicating the number of primers retained at, and timings for, each step.
(EPS)

Table S1 Primers designed to amplify *E. coli* O104:H4 outbreak isolates.
(DOC)

Table S2 Strains used in the experimental validation of predicted diagnostic PCR primers.
(DOC)

Table S3 Experimental validation results for predicted diagnostic primer sets.
(DOC)

Methods S1 (DOC)
