# Supplementary material for: Alignment-Free Design of Highly Discriminatory Diagnostic Primer Sets for Escherichia coli O104:H4 Outbreak Strains
Source: PLoS One. 2012 Apr 5;7(4):e34498. doi: 10.1371/journal.pone.0034498 (PMC3320637; doi:10.1371/journal.pone.0034498)
Supplement: Table S1 — Primers designed to amplify E. coli O104:H4 outbreak isolates. (DOC) [file pone.0034498.s003.doc]

| **Primer Set** | **Location** | **Product**  **Size** | **Primer**  **Length** | **Tm** | **Orientation** | **Sequence** | **Notes** |
| --- | --- | --- | --- | --- | --- | --- | --- |
| **0376** | 357682 | 98 | 20 | 58.98 | forward | TGAAACCACCAAAGCATCAT | Predicted outbreak-specific |
|  | 357760 |  | 20 | 59.07 | reverse | CCAAGGCTGCTACTGAAACA | prophage gp20 transfer protein; not used |
| **0220** | 357687 | 98 | 20 | 59.04 | forward | CCACCAAAGCATCATAAACG | Predicted outbreak-specific |
|  | 357765 |  | 20 | 59.02 | reverse | TGATTCCAAGGCTGCTACTG | prophage gp20 transfer protein; not used |
| **0393** | 357682 | 103 | 20 | 58.98 | forward | TGAAACCACCAAAGCATCAT | Predicted outbreak-specific |
|  | 357765 |  | 20 | 59.02 | reverse | TGATTCCAAGGCTGCTACTG | prophage gp20 transfer protein |
| **0901** | 357968 | 103 | 20 | 58.96 | forward | ACTCGCTTAGGGTCAATGCT | Predicted outbreak-specific |
|  | 358050 |  | 20 | 59.13 | reverse | GGCTAATGAGGGTTCCAAGA | prophage gp20 transfer protein |
| **0781** | 4759179 | 101 | 20 | 59.02 | forward | CCAGAGCATACAGCTTTCCA | Filter: off-target match to O127:H6 (E2348/69) |
|  | 4759260 |  | 20 | 58.82 | reverse | AAGCGATGAAGCTGCTGTTA | hypothetical protein |
| **0396** | 4759843 | 99 | 20 | 58.94 | forward | CCTGCGTATGGCTGAACTTA | Filter: off-target match to O127:H6 (E2348/69) |
|  | 4759922 |  | 20 | 58.91 | reverse | GGAAAGGTAGGGTCGTGGTA | hypothetical protein |
| **0237** | 3708596 | 100 | 20 | 59.08 | forward | AAAGGGAAAGTGTGGTTTGC | Filter: off-target match to β-lactamase plasmid |
|  | 3708676 |  | 20 | 59.09 | reverse | GCAAATCTGTCCATCTGGTG | *impB* on pEC_BacTec plasmid |

**Supplementary Table S1.** Primers designed to amplify *E. coli* O104:H4 outbreak isolates

**Supplementary Table S1.** Primers designed to amplify *E. coli* O104:H4 outbreak isolates. Primer set locations are indicated relative to the GOS2 assembly (Supplementary Methods). Primers 0376 and 0220 (greyed-out) were not used for experimental validation due to their overlap with primer set 0393. Possible off-target matches, and putative annotations for the CDS to which primer sets were designed are indicated in the Notes column.
