# Supplementary material for: Alignment-Free Design of Highly Discriminatory Diagnostic Primer Sets for Escherichia coli O104:H4 Outbreak Strains
Source: PLoS One. 2012 Apr 5;7(4):e34498. doi: 10.1371/journal.pone.0034498 (PMC3320637; doi:10.1371/journal.pone.0034498)
Supplement: Table S2 — Strains used in the experimental validation of predicted diagnostic PCR primers. (DOC) [file pone.0034498.s004.doc]

| **Group 1: Positive Examples** | |  |  |
| --- | --- | --- | --- |
| **Strain no. (*)** | **Serotype** | **clinical diagnosis** | **References** |
| LB226692 | O104:H4 | HUS | Bielaszewska *et al.*, 2011^1^ |
| LB226538 | O104:H4 | HUS | Bielaszewska *et al.*, 2011 |
| LB226542 | O104:H4 | HUS | Bielaszewska *et al.*, 2011 |
| LB226743 | O104:H4 | HUS | Bielaszewska *et al.*, 2011 |
| LB226802 | O104:H4 | HUS | Bielaszewska *et al.*, 2011 |
| LB227019 | O104:H4 | HUS | Bielaszewska *et al.*, 2011 |
| LB227718 | O104:H4 | HUS | Bielaszewska *et al.*, 2011 |
| LB227716 | O104:H4 | Diarrhoea | Bielaszewska *et al.*, 2011 |
| LB226687 | O104:H4 | HUS | Bielaszewska *et al.*, 2011 |
| LB227134 | O104:H4 | HUS | Bielaszewska *et al.*, 2011 |
| LB227103 | O104:H4 | HUS | Bielaszewska *et al.*, 2011 |
| LB227605 | O104:H4 | HUS | Bielaszewska *et al.*, 2011 |
| LB227511 | O104:H4 | Diarrhoea | Bielaszewska *et al.*, 2011 |
| LB227551 | O104:H4 | Bloody diarrhoea | Bielaszewska *et al.*, 2011 |
| LB227695 | O104:H4 | HUS | Bielaszewska *et al.*, 2011 |
| LB227697 | O104:H4 | HUS | Bielaszewska *et al.*, 2011 |
| LB227700 | O104:H4 | Diarrhoea | Bielaszewska *et al.*, 2011 |
| LB227704 | O104:H4 | HUS | Bielaszewska *et al.*, 2011 |
| LB227724 | O104:H4 | HUS | Bielaszewska *et al.*, 2011 |
| LB227726 | O104:H4 | HUS | Bielaszewska *et al.*, 2011 |
| LB227793 | O104:H4 | HUS | Bielaszewska *et al.*, 2011 |
| **Group 2: Negative Examples** | |  |  |
| **Strain no.** | **Serotype** | **HUSEC no.** | **References** |
| 05-946 | O111:H10 | HUSEC001 | Mellmann, 2008, EID^2^ |
| 5152/97 | Ont:H- | HUSEC002 | Mellmann, 2008, EID |
| 6334/96 | O157:H7 | HUSEC003 | Mellmann, 2008, EID |
| 3072/96 | O157:H- | HUSEC004 | Mellmann, 2008, EID |
| 2907/97 | O55:H7 | HUSEC005 | Mellmann, 2008, EID |
| 7382/96 | O103:H2 | HUSEC007 | Mellmann, 2008, EID |
| 1805/00 | O119:H2 | HUSEC010 | Mellmann, 2008, EID |
| 2516/00 | O111:H8 | HUSEC011 | Mellmann, 2008, EID |
| 2245/98 | O26:H11 | HUSEC013 | Mellmann, 2008, EID |
| 1530/99 | O26:H11 | HUSEC018 | Mellmann, 2008, EID |
| 0488/99 | O145:H28 | HUSEC021 | Mellmann, 2008, EID |
| 1169/97/1 | O112:H- | HUSEC023 | Mellmann, 2008, EID |
| 2996/96 | O73:H18 | HUSEC024 | Mellmann, 2008, EID |
| 99-09355 | O113:H21 | HUSEC026 | Mellmann, 2008, EID |
| 03-07727 | O163:H19 | HUSEC027 | Mellmann, 2008, EID |
| 03-06687 | O128:H2 | HUSEC028 | Mellmann, 2008, EID |
| 4256/99 | O70:H8 | HUSEC029 | Mellmann, 2008, EID |
| 05-03519 | O98:H- | HUSEC030 | Mellmann, 2008, EID |
| 7792/96 | OR:H- | HUSEC031 | Mellmann, 2008, EID |
| 2441/98 | O136:Hnt | HUSEC032 | Mellmann, 2008, EID |
| 4392/97 | O145:H25 | HUSEC033 | Mellmann, 2008, EID |
| 3332/99 | O91:H21 | HUSEC034 | Mellmann, 2008, EID |
| 1529/98 | O121:H19 | HUSEC035 | Mellmann, 2008, EID |
| 02-03885 | O104:H21 | HUSEC037 | Mellmann, 2008, EID |
| 3651/96 | O76:H19 | HUSEC039 | Mellmann, 2008, EID |
| 220/00 | O174:H21 | HUSEC040 | Mellmann, 2008, EID |
| 01-09591 | O104:H4 | HUSEC041 | Mellmann, 2008, EID |
| 820/08 | O165:H25 | HUSEC042 | Mellmann, 2008, EID |
| **Strain no.** | **Serotype** | **Pathotype** | **References** |
| 55989 | O104:H4 | EAEC | Muenster collection (**) |
| 2348/69 | O127:H6 (NM) | EPEC (typical) | Iguchi, 2009, J Bact^3^ |
| 6797/96 | O104:H- | EPEC (atypical) | Muenster collection |
| 4823/96 | O104:H21 | EHEC | Muenster collection |

**Supplementary Table S2.** Strains used in the experimental validation of predicted diagnostic PCR primers.

**Supplementary Table S2.** Strains used in the experimental validation of predicted diagnostic PCR primers. Bacterial strains were organised into two groups: group 1 contained O104:H4 isolates from the German 2011 outbreak that were unseen (with the exception of the LB226692 strain) by the primer design process; group 2 contained non-outbreak pathogenic *E. coli* strains that were unseen by the primer design process. The strain numbers, serotype (O-antigen and haemagglutinin), and clinical diagnosis of the source of the isolate are given. A literature reference for each isolate is provided where possible, otherwise the location of the strain collection is indicated.

* all strains were described as referenced, but without strain numbers

** obtained from Erick Denamur, INSERM, France

1. Bielaszewska, M. et al. Characterisation of the Escherichia coli strain associated with an outbreak of haemolytic uraemic syndrome in Germany, 2011: a microbiological study. *Lancet Infect Dis* **11**, 671–676 (2011).

2. Mellmann, A. et al. Analysis of collection of hemolytic uremic syndrome-associated enterohemorrhagic Escherichia coli. *Emerging Infect Dis* **14**, 1287–1290 (2008).

3. Iguchi, A. et al. Complete genome sequence and comparative genome analysis of enteropathogenic Escherichia coli O127:H6 strain E2348/69. *J Bacteriol* **191**, 347–354 (2009).
