# Supplementary material for: Alignment-Free Design of Highly Discriminatory Diagnostic Primer Sets for Escherichia coli O104:H4 Outbreak Strains
Source: PLoS One. 2012 Apr 5;7(4):e34498. doi: 10.1371/journal.pone.0034498 (PMC3320637; doi:10.1371/journal.pone.0034498)
Supplement: Table S3 — Experimental validation results for predicted diagnostic primer sets. (DOC) [file pone.0034498.s005.doc]

|  |  |  |  | **Primer sets** | | |  |  |
| --- | --- | --- | --- | --- | --- | --- | --- | --- |
| **Number** | **My names** | **Strain no.** | **Serotypes/Clin diag** | **393** | **901** | **781** | **396** | **237** |
| 1 | L1 | LB226692 | HUS | Y | Y | Y | Y | Y |
| 2 | L2 | LB226538 | HUS | Y | Y | Y | Y | Y |
| 3 | L4 | LB226542 | HUS | Y | Y | Y | Y | Y |
| 4 | L6 | LB226743 | HUS | Y | Y | Y | Y | Y |
| 5 | L12 | LB226802 | HUS | Y | Y | Y | Y | Y |
| 6 | L15 | LB227019 | HUS | Y | Y | Y | Y | Y |
| 7 | L17 | LB227718 | HUS | Y | Y | Y | Y | Y |
| 8 | L19 | LB227716 | Diarrhoea | Y | Y | Y | Y | Y |
| 9 | L20 | LB226687 | HUS | Y | Y | Y | Y | Y |
| 10 | L22 | LB227134 | HUS | Y | Y | Y | Y | Y |
| 11 | L23 | LB227103 | HUS | Y | Y | Y | Y | Y |
| 12 | L31 | LB227605 | HUS | Y | Y | Y | Y | Y |
| 13 | L33 | LB227511 | Diarrhoea | Y | Y | Y | Y | Y |
| 14 | L34 | LB227551 | Bloody diarrhoea | Y | Y | Y | Y | Y |
| 15 | L46 | LB227695 | HUS | Y | Y | Y | Y | Y |
| 16 | L47 | LB227697 | HUS | Y | Y | Y | Y | Y |
| 17 | L48 | LB227700 | Diarrhoea | Y | Y | Y | Y | Y |
| 18 | L50 | LB227704 | HUS | Y | Y | Y | Y | Y |
| 19 | L51 | LB227724 | HUS | Y | Y | Y | Y | Y |
| 20 | L52 | LB227726 | HUS | Y | Y | Y | Y | Y |
| 21 | L54 | LB227793 | HUS | Y | Y | Y | Y | Y |
| 1 | O1 | 55989 | O104:H4 | N | N | N | N | N |
| 2 | O2 | 2348/69 | O127:H6 (NM) | N | N | Y | Y | N |
| 3 | O3 | 6797/96 | O104:H- | N | Y | N | N | Y |
| 4 | O4 | 4823/96 | 0104:H21 | N | N | N | N | Y |
| 5 | H1 | HUSEC001 | O111:H10 | Y | Y | N | N | N |
| 6 | H2 | HUSEC002 | Ont:H- | N | N | N | N | N |
| 7 | H3 | HUSEC 003 | O157:H7 | N | N | N | N | N |
| 8 | H4 | HUSEC004 | O157:H- | N | N | N | N | N |
| 9 | H5 | HUSEC005 | O55:H7 | N | N | N | N | N |
| 10 | H7 | HUSEC007 | O103:H2 | N | N | N | N | N |
| 11 | H10 | HUSEC010 | O119:H2 | N | N | N | N | N |
| 12 | H11 | HUSEC011 | O111:H8 | N | N | N | N | Y |
| 13 | H13 | HUSEC013 | O26:H11 | N | N | N | N | N |
| 14 | H18 | HUSEC018 | O26:H11 | N | N | N | N | N |
| 15 | H21 | HUSEC021 | O145:H28 | N | N | N | N | N |
| 16 | H23 | HUSEC023 | O112:H- | N | N | N | N | N |
| 17 | H24 | HUSEC024 | O73:H18 | N | N | N | N | N |
| 18 | H26 | HUSEC026 | O113:H21 | N | N | N | N | N |
| 19 | H27 | HUSEC027 | O163:H19 | N | N | N | N | N |
| 20 | H28 | HUSEC028 | O128:H2 | N | N | N | N | N |
| 21 | H29 | HUSEC029 | O70:H8 | N | N | N | N | N |
| 22 | H31 | HUSEC030 | O98:H- | N | N | N | N | N |
| 23 | H31 | HUSEC031 | OR:H- | Y | Y | N | N | N |
| 24 | H32 | HUSEC032 | O136:Hnt | N | N | N | N | Y |
| 25 | H33 | HUSEC033 | O145:H25 | N | N | N | N | N |
| 26 | H34 | HUSEC034 | O91:H21 | N | N | N | N | N |
| 27 | J35 | HUSEC035 | O121:H19 | N | N | N | N | Y |
| 28 | J37 | HUSEC037 | O104:H21 | N | N | N | N | N |
| 29 | H39 | HUSEC039 | O76:H19 | N | N | N | N | N |
| 30 | H40 | HUSEC040 | O174:H21 | N | N | N | N | Y |
| 31 | H41 | HUSEC041 | O104:H4 | Y | Y | Y | Y | N |
| 32 | H42 | HUSEC042 | O165:H25 | N | N | N | N | N |
| 1 | control -ve |  |  | N | N | N | N | N |

**Supplementary Table S3.** Experimental validation results for predicted diagnostic primer sets.

**Supplementary Table S3.** Experimental validation results for predicted diagnostic primer sets. Group 1 isolates L1-L54 are positive examples of O104:H4 outbreak strains unseen by the primer design process (except for positive control L1), and group 2 isolates (O1-O4; H1-H42) are negative examples of non-outbreak strains unseen by the primer design process. Amplification of an isolate by each primer set is indicated by the letter ‘Y’ against a yellow background, and the failure to amplify is indicated by the letter ‘N’ against a red background. All group 1 isolates are amplified by all primer sets.
