# Supplementary material for: Alignment-Free Design of Highly Discriminatory Diagnostic Primer Sets for Escherichia coli O104:H4 Outbreak Strains
Source: PLoS One. 2012 Apr 5;7(4):e34498. doi: 10.1371/journal.pone.0034498 (PMC3320637; doi:10.1371/journal.pone.0034498)
Supplement: Methods S1 — (DOC) [file pone.0034498.s006.doc]

## SUPPLEMENTARY METHODS

## Primer Prediction Pipeline: Analysis details and default settings

The bioinformatic pipeline outlined schematically in Figure 1 and Supplementary Figure 1 was implemented in a Python script with multiple options that are configurable by the user, and uses the Biopython software libraries[1], EMBOSS software packages ePrimer3 and primersearch {Rozen:2000wg, Rice:2000wr}, the CDS prediction package prodigal[4], and NCBI BLAST[5]. The essential steps of running this pipeline are enumerated below:

1. The user generates a configuration file associating genome abbreviations with the locations of sequence data, and a classification (e.g. species), or list of classifications for each set of sequence data.
2. The user runs the find_differential_primers.py script that, for each input genome, carries out the following steps:
   1. If the genome is a draft assembly of several contigs, concatenate all contigs into a single sequence using a separator sequence with in-frame start and stop codons for all six frames[6]: NNNNNCATTCCATTCATTAATTAATTAATGAATGAATGNNNNN, writing this to a new location on disk.
   2. If primers are to be filtered on whether they’re located within CDS features (a user-configurable setting), predicted CDS locations for the genome are loaded from file if this is given in the configuration file. If existing feature locations are not specified, CDS predictions for the sequence are generated using prodigal[4].
   3. If existing primers are provided in ePrimer3 format in the configuration file, these are loaded. Otherwise eprimer3[3] is run to generate a set of primer candidates (and internal oligos, if specified) on the genome.
   4. If required by the user, predicted primers are filtered on the presence of GC at the 3` end and other characteristics. Primer sets are tagged if they match a filter.
   5. If existing BLAST+ screen results are provided in the configuration file, these are loaded. Otherwise candidate primers are used as BLAST+ queries against screening sequences in a named database. Primer sets are tagged if they match in the screen.
   6. If existing primersearch output is not provided in the configuration file, primer candidates are written to primersearch input format, and primersearch[3] run with these candidates as input on each other genome/assembly sequence in turn.
   7. primersearch results are read in, and each primer set tagged with the organisms that it cross-amplifies.
   8. If classification is required by the user, all tags are examined, and primer sets partitioned according to whether, in addition to meeting thermodynamic and sequence criteria and any user-specified BLAST screen, they are classification-specific, sequence-specific, or universal to all input sequences.
   9. Summary data, amplicons and sets of primers are written to plain text output files, grouped by category: classification-specific, sequence-specific, and ’universal’.

## Gene boundary prediction with prodigal

If the --nocds option is set to true when running the pipeline script, then no filtering of primers on the basis of location within CDS features is carried out; the default setting is to filter for primers located within CDS features.

If a GenBank format feature file is specified for a sequence in the configuration file, this is used as the source for CDS locations. Otherwise, so long as the --noprodigal option is not set, CDS boundaries are obtained for each draft assembly using prodigal (v1.20 for this study)[4], with the protocol

prodigal -a output.features < chromosome.fas > output.prodigalout

This protocol takes a FASTA format sequence file representing a complete chromosome (chromosome.fas) as input, and produces a GenBank-like output file describing CDS locations in the file output.prodigalout. It also produces a FASTA format file of conceptual CDS translations in output.features.

## Primer pair prediction with eprimer3

If an existing ePrimer3 file is specified for a sequence in the configuration file, its contents are used as the primer set for that organism. Otherwise, so long as the --noprimer3 option is not set to true, the EMBOSS package ePrimer3[3] is used to predict candidate primer sets for each sequence. If the --hybridprobe option is set to true, then an internal oligo is also predicted for each primer set; setting this option will generally modify the returned primer set as not all thermodynamically acceptable primer sets produce amplicons containing a thermodynamically acceptable amplicon.

ePrimer3 is run with the following default parameters, which may also be modified on the pipeline command-line:

- numreturn: 20
- osize: 20
- minsize: 18
- maxsize: 22
- otm: 59
- mintm: 58
- maxtm: 60
- ogcpercent: 55
- mingc: 30
- maxgc: 80
- psizeopt: 100
- psizemin: 50
- psizemax: 150
- maxpolyx: 3
- hybridprobe: False
- oligoosize: 20
- oligominsize: 13
- oligomaxsize: 30
- oligootm: 69
- oligomintm: 68
- oligomaxtm: 70
- oligoogcpercent: 55
- oligomingc: 30
- oligomaxgc: 80
- oligomaxpolyx: 3

Predicted primer sets are loaded into the pipeline, and can be tagged according to whether or not they pass certain filters. The primer sets can then be written out to file according to whether user-defined combinations of filters have been passed or not, as described below.

## Sequence screening of primers with BLAST+

The BLAST+ package[5] is used to screen predicted primers against a specified database if the --blastdb option is set with the location of a suitable BLAST database. Alternatively, the --useblastoption can be set to True, so that the pipeline uses precalculated BLAST XML output.

In the BLAST screen, forward and reverse primers from each set are used as individual queries against the specified database. If either primer makes a suitable match (greater than 90% sequence identity over the full length of the query primer sequence) to the database, the primer set is tagged as making a match in the database. The BLAST+ parameters, which cannot be modified on the pipeline script command-line, are:

- task: ’blastn’
- num_alignments: 1
- num_descriptions: 1
- outfmt: 5
- perc_identity: 90
- ungapped: True

## Filtering primer pairs with primersearch

If the --noprimersearch option is set to True, the pipeline loads in all primersearch output it can find in the same directory as the source sequences. If this data is incomplete for all-against-all pairwise comparisons, an error will be thrown. If the --noprimersearch option is set to False, primersearch[3] is carried out in an all-against-all analysis of each primer set from each sequence against all other sequences, to identify potential cross-amplification, with the following protocol:

primersearch -seqall target.fas -infile primers.primers -outfile output.primersearch -mismatchpercent 10

The mismatchpercent parameter is modifiable at the command-line.

## Distributed processing

Some individual stages in the pipeline are relatively slow, but are applicable almost identically to several input data sets. We use the Python multiprocessing module to parallelise these tasks by distributing them across available processors, speeding up the pipeline several-fold. By default, all available processors are used, but this number can be modified at the command-line. This requires the Python multiprocessing module, present in Python versions 2.6 and later.

**Input Sequences**

The sequences used in the ‘positive’ training set were eleven draft assemblies of the nine *E. coli* O104:H4 German outbreak isolates, downloaded from the *E. coli* O104:H4 Genome Analysis Crowdsourcing site (<https://github.com/ehec-outbreak-crowdsourced/BGI-data-analysis/wiki>) on 27/6/2011[7]:

- TY2482 (two assemblies: BGI, Universal Medical Centre Hamburg-Eppendorf, Germany)
- LB226692 (two assemblies: Life Tech, University Hospital Muenster, Germany)
- H112180280, H112240282, H112240283, H112240540, H112240541 (HPA, Colindale, UK)
- GOS1, GOS2 (Goettingen Genomics Lab, Germany)

The sequences used in the ‘negative’ training set were the completely sequenced *E. coli* and *E. fergusonii* genomes available from GenBank (ftp://ftp.ncbi.nlm.nih.gov/genomes/Bacteria/), downloaded at 27/6/2011[8]:

- *Escherichia coli* str. K-12 substr. W3110 chromosome, complete genome (AC_000091)
- *Escherichia coli* str. K-12 substr. MG1655 chromosome, complete genome (NC_000913_
- *Escherichia coli* O157:H7 str. Sakai plasmid pOSAK1, complete sequence (NC_002127)
- *Escherichia coli* O157:H7 str. Sakai plasmid pO157, complete sequence (NC_002128)
- *Escherichia coli* O157:H7 str. EDL933 chromosome, complete genome (NC_002655)
- *Escherichia coli* O157:H7 str. Sakai, complete genome (NC_002695)
- *Escherichia coli* CFT073, complete genome (NC_004431)
- *Escherichia coli* O157:H7 EDL933 plasmid pO157, complete sequence (NC_007414)
- *Escherichia coli* UTI89 plasmid pUTI89, complete sequence (NC_007941)
- *Escherichia coli* UTI89, complete genome (NC_007946)
- *Escherichia coli* 536, complete genome (NC_008253)
- *Escherichia coli* APEC O1, complete genome (NC_008563)
- *Escherichia coli* E24377A plasmid pETEC_80, complete sequence (NC_009786)
- *Escherichia coli* E24377A plasmid pETEC_35, complete sequence (NC_009787)
- *Escherichia coli* E24377A plasmid pETEC_73, complete sequence (NC_009788)
- *Escherichia coli* E24377A plasmid pETEC_6, complete sequence (NC_009789)
- *Escherichia coli* E24377A plasmid pETEC_74, complete sequence (NC_009790)
- *Escherichia coli* E24377A plasmid pETEC_5, complete sequence (NC_009791)
- *Escherichia coli* HS, complete genome (NC_009800)
- *Escherichia coli* E24377A, complete genome (NC_009801)
- *Escherichia coli* APEC O1 plasmid pAPEC-O1-ColBM, complete sequence (NC_009837)
- *Escherichia coli* APEC O1 plasmid pAPEC-O1-R, complete sequence (NC_009838)
- *Escherichia coli* ATCC 8739, complete genome (NC_010468)
- *Escherichia coli* str. K-12 substr. DH10B, complete genome (NC_010473)
- *Escherichia coli* SMS-3-5 plasmid pSMS35_8, complete sequence (NC_010485)
- *Escherichia coli* SMS-3-5 plasmid pSMS35_4, complete sequence (NC_010486)
- *Escherichia coli* SMS-3-5 plasmid pSMS35_3, complete sequence (NC_010487)
- *Escherichia coli* SMS-3-5 plasmid pSMS35_130, complete sequence (NC_010488)
- *Escherichia coli* SMS-3-5, complete genome (NC_010498)
- *Escherichia coli* O157:H7 str. EC4115 plasmid pO157, complete sequence (NC_011350)
- *Escherichia coli* O157:H7 str. EC4115 plasmid pEC4115, complete sequence (NC_011351)
- *Escherichia coli* O157:H7 str. EC4115, complete genome (NC_011353)
- *Escherichia coli* SE11 plasmid pSE11-4, complete sequence (NC_011407)
- *Escherichia coli* SE11 plasmid pSE11-5, complete sequence (NC_011408)
- *Escherichia coli* SE11 plasmid pSE11-6, complete sequence (NC_011411)
- *Escherichia coli* SE11 plasmid pSE11-2, complete sequence (NC_011413)
- *Escherichia coli* SE11, complete genome (NC_011415)
- *Escherichia coli* SE11 plasmid pSE11-3, complete sequence (NC_011416)
- *Escherichia coli* SE11 plasmid pSE11-1, complete sequence (NC_011419)
- *Escherichia coli* UMN026 plasmid p2ESCUM, complete sequence (NC_011739)
- *Escherichia fergusonii* ATCC 35469, complete genome (NC_011740)
- *Escherichia coli* IAI1, complete genome (NC_011741)
- *Escherichia coli* S88, complete genome (NC_011742)
- *Escherichia fergusonii* ATCC 35469 plasmid pEFER, complete sequence (NC_011743)
- *Escherichia coli* ED1a, complete genome (NC_011745)
- *Escherichia coli* S88 plasmid pECOS88, complete sequence (NC_011747)
- *Escherichia coli* 55989, complete genome (NC_011748)
- *Escherichia coli* UMN026 plasmid p1ESCUM, complete sequence (NC_011749)
- *Escherichia coli* IAI39, complete genome (NC_011750)
- *Escherichia coli* UMN026, complete genome (NC_011751)
- *Escherichia coli* BW2952, complete genome (NC_012759)
- *Escherichia coli* BL21-Gold(DE3)pLysS AG chromosome, complete genome (NC_012947)
- *Escherichia coli* B str. REL606 chromosome, complete genome (NC_012967)
- *Escherichia coli* O157:H7 str. TW14359, complete genome (NC_013008)
- *Escherichia coli* O157:H7 str. TW14359 plasmid pO157, complete sequence (NC_013010)
- *Escherichia coli* O103:H2 str. 12009, complete genome (NC_013353)
- *Escherichia coli* O103:H2 str. 12009 plasmid pO103, complete sequence (NC_013354)
- *Escherichia coli* O26:H11 str. 11368, complete genome (NC_013361)
- *Escherichia coli* O26:H11 str. 11368 plasmid pO26_2, complete sequence (NC_013362)
- *Escherichia coli* O26:H11 str. 11368 plasmid pO26_3, complete sequence (NC_013363)
- *Escherichia coli* O111:H- str. 11128, complete genome (NC_013364)
- *Escherichia coli* O111:H- str. 11128 plasmid pO111_1, complete sequence (NC_013365)
- *Escherichia coli* O111:H- str. 11128 plasmid pO111_3, complete sequence (NC_013366)
- *Escherichia coli* O111:H- str. 11128 plasmid pO111_4, complete sequence (NC_013367)
- *Escherichia coli* O111:H- str. 11128 plasmid pO111_5, complete sequence (NC_013368)
- *Escherichia coli* O26:H11 str. 11368 plasmid pO26_1, complete sequence (NC_013369)
- *Escherichia coli* O111:H- str. 11128 plasmid pO111_2, complete sequence (NC_013370)
- *Escherichia coli* O55:H7 str. CB9615 chromosome, complete genome (NC_013941)
- *Escherichia coli* O55:H7 str. CB9615 plasmid pO55, complete sequence (NC_013942)
- *Escherichia coli* O26:H11 str. 11368 plasmid pO26_4, complete sequence (NC_014543)

## Sequences included in the BLAST screening database

The refseq_genomic database sequences that lay beneath the *Escherichia* taxonomic level (TaxID: 561) at the NCBI on 6/7/2011 were used as the online screening database for the seven primers predicted to be diagnostic (Supplementary Table S1).

**Continued monitoring of primer specificity**

The predicted specificity of diagnostic primers is critically dependent on the composition of the training and screening sequence sets. These datasets are necessarily incomplete, as they do not contain sequences for every isolate that is known or may be encountered in the field. The rate of addition of complete and draft sequences to the public databases is however very rapid, and offers an opportunity for continued monitoring of primer specificity *in silico* as new sequences become publicly available. Identification of potential off-target amplification against newly published sequences *in silico* may be automated to flag a need for revision of primer specificity, and possible primer redesign.

Subsequent to the experimental validation of the predicted primers in this study, the complete sequence of a novel *E. coli* isolate (UMNF18; GenBank CP002890.1) that was unavailable during our training and validation process was entered into the public repositories. This genome exhibits *in silico* potential off target amplification by the four primers that target the gp20 transfer sequence (0220, 0376, 0393, and 0901), and highlights the necessity for continued monitoring of primer specificity as new sequences become available.

**Experimental Validation**

**Bacteria**

Specificity of the predicted PCR primer sets was validated experimentally using 53 *E. coli* clinical isolates (Supplementary Table S2). Group 1 comprised 21 outbreak isolates collected from Universitätsklinikum Münster; group 2 comprised 28 haemolytic uraemic syndrome (HUSEC) isolates from the Universitätsklinikum Münster collection[9] and four additional EPEC and/or HUSEC isolates from elsewhere[10] and from the Universitätsklinikum Münster collection. Bacteria in Group 1 were isolated from stool samples from patients exhibiting clinical symptoms of hæmolytic uræmic syndrome (HUS) and/or diarrhoea and identified as *E. coli* O104:H4[11]. The majority of *E. coli* isolates in Group 2 belong to the characterised HUSEC collection[9] and the remainder are representatives of atypical enteropathogenic *E. coli* (EPEC), a characterised EPEC and an enteroaggregative *E. coli* (EAEC). Bacteria were cultured in LB broth at 37^o^C, with aeration, and cryoprotected at -80°C.

**DNA extraction and purification**

Genomic DNA was prepared from a single bacterial colony, using Instagene Matrix, a Chelex–based resin for 'PCR-ready' DNA, as described by the manufacturer, (Bio-Rad).

**PCR evaluation**

Primer sequences:

| Primer set | Primer name | Sequence | Product size (bp) |
| --- | --- | --- | --- |
| 0393 | 0393_FOR | 5'-TGAAACCACCAAAGCATCAT | 103 |
|  | 0393_REV | 5'-TGATTCCAAGGCTGCTACTG |  |
| 0901 | 0901_FOR | 5'-ACTCGCTTAGGGTCAATGCT | 102 |
|  | 0901_REV | 5'-GGCTAATGAGGGTTCCAAGA |  |
| 0781 | 0781_FOR | 5'-CCAGAGCATACAGCTTTCCA | 101 |
|  | 0781_REV | 5'-AAGCGATGAAGCTGCTGTTA |  |
| 0396 | 0396_FOR | 5'-CCTGCGTATGGCTGAACTTA | 99 |
|  | 0396_REV | 5'-GGAAAGGTAGGGTCGTGGTA |  |
| 0237 | 0237_FOR | 5'-AAAGGGAAAGTGTGGTTTGC | 100 |
|  | 0237_REV | 5'-GCAAATCTGTCCATCTGGTG |  |

The primer sets formed two groups on the basis of *in silico* prediction:

(a) primers 0393 and 0901 were expected to amplify only sequences restricted to *E. coli* O104:H4 from the 2011 outbreak, and not to amplify sequences from other *E. coli* pathotypes

(b) primers 0781, 0396 and 0237 were expected, on the basis of filtering against RefSeq[12] to target sequences from an extended set of *E. coli* isolates including all *E. coli* O104:H4 2011 outbreak isolates and some other related *E. coli* pathotypes.

Two groups of bacteria were selected for PCR validation:

(i) Group 1 isolates constitute a positive set of examples unseen during the primer design process, and were derived from the 2011 German outbreak of *E. coli* O104:H4. These isolates were expected to be clonally related to *E. coli* LB226692. It was anticipated that the complete primer set would target sequences in all outbreak isolates.

(ii) Group 2 isolates constitute a negative set of examples unseen during the primer design process, and were selected to represent a broad range of *E. coli* pathotypes (EPEC, HUSEC, EAEC) and serotypes, distinct from the 2011 German outbreak isolates. Group 2 also included historical isolates with the same O104:H4 serotype (e.g. *E. coli* HUSEC041 and 55989). Further isolates, (e.g. *E. coli* 2348/69) were included that were expected, on the basis of RefSeq searches, to contain target sequences for 0781, 0396 or 0237.

**PCR conditions**

The optimum annealing temperature for the complete primer set first determined to be 58^o^C using the sequenced *E. coli* isolate LB226692 as a positive control between 54^o^C and 58^o^C. PCR reactions of 25 ml contained 500 nM of each primer pair, 800 mM dNTPs, 1 x buffer solution, 1 x enhancer solution, 2 mM MgCl_2_, 0.5 units/µL peqGOLD *Taq* polymerase (peqlab) and 1 ml DNA (~ 100 pg). Standard cycling conditions were used: one cycle of 94^o^C for 5 minutes; 30 cycles of 94^o^C for 30 seconds, 58^o^C for 1 minute, 72^o^C for 1 minute; one cycle of 72^o^C for five minutes. Products were resolved on a 1 % agarose gel in TAE buffer, alongside a 100 bp ladder. The images were captured in a gel documentation system (AlphaImager, Labtech) at 300 dpi and saved in TIFF format. Image manipulations were carried out using Adobe Illustrator.

**References**

1. Cock PJA, Antao T, Chang JT, Chapman BA, Cox CJ, et al. (2009) Biopython: freely available Python tools for computational molecular biology and bioinformatics. Bioinformatics 25: 1422–1423. doi:10.1093/bioinformatics/btp163

2. Rozen S, Skaletsky H (2000) Primer3 on the WWW for general users and for biologist programmers. Methods Mol Biol 132: 365–386.

3. Rice P, Longden I, Bleasby A (2000) EMBOSS: The European molecular biology open software suite. Trends Genet 16: 276–277.

4. Hyatt D, Chen G-L, Locascio PF, Land ML, Larimer FW, et al. (2010) Prodigal: prokaryotic gene recognition and translation initiation site identification. BMC Bioinformatics 11: 119. doi:10.1186/1471-2105-11-119

5. Camacho C, Coulouris G, Avagyan V, Ma N, Papadopoulos J, et al. (2009) BLAST+: architecture and applications. BMC Bioinformatics 15: 421.

6. Reinhardt JA, Baltrus DA, Nishimura MT, Jeck WR, Jones CD, et al. (2009) De novo assembly using low-coverage short read sequence data from the rice pathogen Pseudomonas syringae pv. oryzae. Genome Res 19: 294–305. doi:10.1101/gr.083311.108

7. E. coli O104:H4 Genome Analysis Crowdsourcing - GitHub (n.d.) E. coli O104:H4 Genome Analysis Crowdsourcing - GitHub. github.com. Available: https://github.com/ehec-outbreak-crowdsourced/BGI-data-analysis/wiki. Accessed 27 Jun. 2011.

8. NCBI Bacterial Genome Sequence FTP Index (n.d.) NCBI Bacterial Genome Sequence FTP Index. ncbi.nlm.nih.gov. Available: ftp://ftp.ncbi.nlm.nih.gov/genomes/Bacteria. Accessed 6 Sep. 2011.

9. Mellmann A, Bielaszewska M, Köck R, Friedrich AW, Fruth A, et al. (2008) Analysis of collection of hemolytic uremic syndrome-associated enterohemorrhagic Escherichia coli. Emerging Infect Dis 14: 1287–1290. doi:10.3201/eid1408.071082

10. Iguchi A, Thomson NR, Ogura Y, Saunders D, Ooka T, et al. (2009) Complete genome sequence and comparative genome analysis of enteropathogenic Escherichia coli O127:H6 strain E2348/69. J Bacteriol 191: 347–354. doi:10.1128/JB.01238-08

11. Bielaszewska M, Mellmann A, Zhang W, Köck R, Fruth A, et al. (2011) Characterisation of the Escherichia coli strain associated with an outbreak of haemolytic uraemic syndrome in Germany, 2011: a microbiological study. Lancet Infect Dis 11: 671–676. doi:10.1016/S1473-3099(11)70165-7

12. NCBI RefSeq Database (n.d.) NCBI RefSeq Database. Available: http://www.ncbi.nlm.nih.gov/RefSeq. Accessed 6 Sep. 2011.
